# Coordinated Regulation of Chromatophore Differentiation and Melanogenesis during the Ontogeny of Skin Pigmentation of *Solea senegalensis* (Kaup, 1858)

**DOI:** 10.1371/journal.pone.0063005

**Published:** 2013-05-09

**Authors:** Maria J. Darias, Karl B. Andree, Anaïs Boglino, Ignacio Fernández, Alicia Estévez, Enric Gisbert

**Affiliations:** 1 Centre de Sant Carles de la Ràpita, Unitat de Cultius Experimentals, Institut de Recerca i Tecnologia Agroalimentàries, Sant Carles de la Ràpita, Catalònia, Spain; 2 Evolutionary and Developmental Gene Expression Department, Centro de Ciências do Mar, Universidade do Algarve, Faro, Portugal; University of Massachusetts, United States of America

## Abstract

Abnormal pigmentation of Senegalese sole has been described as one problem facing the full exploitation of its commercial production. To improve our understanding of flatfish pigmentation of this commercially important species we have evaluated eleven genes related to two different processes of pigmentation: melanophore differentiation, and melanin production. The temporal distribution of gene expression peaks corresponds well with changes in pigmentation patterns and the intensity of skin melanization. Several gene ratios were also examined to put in perspective possible genetic markers for the different stages of normal pigmentation development. Further, the phenotypic changes that occur during morphogenesis correspond well with the main transitions in gene expression that occur. Given the dramatic phenotypic alterations which flatfish undergo, including the asymmetric coloration that occurs between the ocular and the blind side, and the synchrony of the two processes of morphogenesis and pigmentation ontogenesis, these species constitute an interesting model for the study of pigmentation. In this study we present a first approximation towards explaining the genetic mechanisms for regulating pigmentation ontogeny in Senegalese sole, *Solea senegalensis*.

## Introduction

Skin pigmentation of fishes is the result of the spatial combination and changes in number of several types of chromatophores that produce a huge variety of pigment patterns contributing to sex recognition, camouflage and predator avoidance, and speciation [1], [2], [3]. These neural crest-derived pigment cells are dermal and epidermal dark (brown-black) colored melanophores (equivalent to mammal melanocytes), yellow-orange xanthophores, red erythrophores, iridescent iridophores, white leucophores and blue cyanophores [4]. However, little is known about how these patterns are generated [5], [6]. Knowledge of the molecular ontogeny and pigment cell behavior underlying skin coloring is an essential step in understanding not only the origins of naturally occurring trait variation and evolution [7], but also the pigmentation disorders appearing in later stages of development [8], [9]. Insights into the mechanisms underlying these patterns can be gained by analyzing the expression profiles of pigmentation-related genes during the larval development of the fish.

Pigmentation of flatfish has been a subject of special interest since the 19th century because of the remarkable capacity to change skin color to mimic texture and color of the background [10], [11], [12], [13]. The first works were devoted to the study of skin morphology [14], the type of pigments of the skin and their location in tissues. The studies of Burton since 1975 until present, which were more focused on the pleuronectids, have contributed greatly to the knowledge of the chromatic biology and physiology of chromatophores associated to changes in color pattern [15], [16], [17]. Another notable characteristic of flatfish is that they undergo a complex process of metamorphosis during development that comprises profound morphological and physiological changes associated with eye migration, a 90° rotation in body and asymmetrical pigmentation [18]. After metamorphosis, the most common chromatophores on the blind side of flatfish are the iridophores, whereas on the ocular side there are melanophores and iridophores. The final color of the skin is determined by the amount and distribution of both types of chromatophores [17].

The process of metamorphosis in fish is mirrored in the molecular features [18], [19], [20], [21] and changes in gene expression patterns during metamorphosis are necessary to progress from the larval to the adult phenotype. Indeed, malpigmentation in flatfish seems to be the result of a disruption of the development of pigment cells at metamorphosis [8], [9]. Flatfish can develop pigmentation abnormalities under intensive rearing conditions, which makes them suitable models for the study of the origin of pigmentation disorders. Several environmental factors have shown to induce pigmentation problems, especially related to imbalanced nutrition [22], [23], [24], [25], [26]. For instance, it has been shown that excessive amounts of dietary arachidonic acid during the larval development of Senegalese sole, *Solea senegalensis* (Kaup, 1858) could induce up to 90% of pseudo-albinism [25]. To our knowledge, there is no information about the ontogeny of pigmentation in this species, although the analysis of the temporal and spatial distribution of chromatophores is essential to elucidate the mechanism of formation of the adult pigmentation pattern [27]. Moreover, little is known about the molecular mechanisms underlying pigmentation processes in flatfish, including pigmentation disorders; most of the information found being very recent [27] or coming from medaka (*Oryzias latipes*) and zebrafish (*Danio rerio*), and referring to a single or few genes [28], [29], [30].

The aim of this work was to 1) describe the morphological development of skin pigmentation and 2) analyze the expression profile of eleven genes involved in melanophore differentiation and melanin synthesis during larval development of Senegalese sole (*Solea senegalensis*).

These genes and their protein products are: I) The melanocyte-stimulating hormone 1 receptor (*mc1r*), which is the “classical” receptor of the *α*-melanocyte-stimulating hormone (*α-MSH*) [31] and has a key role in determining the pigmentation of skin and hair in mammals [32], [33]. In fish, *mc1r* is involved in skin color changes [4] and its role in the pigmentation pattern during development has been recently reported [7]. II) The agouti signaling protein (*asip*), which in mammals regulates the relative proportions of eumelanin (black-brown pigment) and phaeomelanin (yellow-red pigment) by antagonizing the action of *α*-MSH on its receptor MC1R [34]. In fish, it has been shown that *asip* is abundantly expressed in the ventral skin, but scarcely in dorsal skin [35], demonstrating the involvement of *α-MSH* and *asip* in the assignment of a dorsal–ventral pigment pattern in fish. III) The paired box protein 3 (*pax3*), a key transcription factor for influencing the development of the neural crest and neural crest-derivatives during mammalian embryogenesis, and that influences melanocytic proliferation, resistance to apoptosis, migration, lineage specificity and differentiation [36]. *Pax3* both promotes and inhibits melanogenesis within these cells through transcriptional regulation of microphtalmia-associated transcription factor (*mitf*), L-dopachrome tautomerase (*dct/trp2*), tyrosinase-related protein 1 (*trp1*) [37] and the mast/stem cell growth factor receptor Kit, *cKit* [38]. In zebrafish, *pax3* is required for fate specification of xanthophores and for melanophore development [39]. IV) The protein product of *mitf,* one the earliest genes expressed in melanoblast precursors [40], which has an essential role in the differentiation and proliferation of melanocytes/melanophores. It is considered the master regulator of melanogenesis due to its ability to activate many melanocyte-specific genes, such as tyrosinase (*tyr*), *trp1* and *dct* [30], [41]. V) The protein product of *cKit,* which plays a critical role in melanocyte physiology by influencing melanogenesis, proliferation, migration, and survival of these cells [42]. In zebrafish, *cKit* promotes the processes of larval melanophore migration and survival [43]. Activation of *cKit* induces *tyr* gene transcription and melanin synthesis in differentiated cultured melanocytes [44]. VI) The *tyr* gene, which codes for tyrosinase, the first enzyme of the biosynthetic pathway of melanin that oxidizes the amino acid L-tyrosine to dopaquinone [45]. VII) The *trp1* gene, coding for the last enzyme of melanogenesis in mice, which catalyzes the oxidation of indolic intermediate 5,6-dihydroxyindole-2-carboxylic acid (DHICA) to produce eumelanin [46]. In humans, together with TRP1, TYR can also act at the last step of the melanin synthesis [47]. The function of *trp1* in fish is scarcely known, although it seems that its presence is necessary for the formation of melanin ([48], Darias et al., unpublished data). *Trp1* has been cloned in several fish species [49] but its expression has not been studied during larval development. VIII) The sodium/potassium/calcium exchanger 5 (*slc24a5*), a putative cation exchanger localized to intracellular membranes of melanosomes and their precursors. Its mRNA was first isolated in zebrafish and it is believed to increase uptake and accumulation of calcium in melanophores, necessary for melanogenesis [50]. It has been recently demonstrated that *slc24a5* is important for normal melanization processes in all pigmented cells of mice [51]. Moreover, it has been suggested to be involved in the development of ocular albinism and macular degeneration in humans and pseudo-albinism in flatfish ([51], Darias et al., unpublished data). IX) The enzyme caspase 3 (*casp3*), involved in the activation cascade of caspases responsible for execution of apoptosis [52]. In humans, *casp3* is able to cleave *mitf*, thus conferring proapoptotic functions to this gene to modulate death in melanocytes and melanoma cells [53]. No reports have been found regarding the natural influence of this gene in the physiology of melanophores in fish. X) The heat shock 70 kDa protein (*hsp70*), a stress protein that confers cell protection against stressors that is able to suppress melanin production in a mouse melanoma cell line (B16) and in the epidermis of mice through the down-regulation of *tyr* [54], [55]. XII) The somatolactin gene (*sl*), a fish-specific peptide hormone secreted from the pituitary gland [56] and, regarding pigmentation, is involved in adaptation to background in red drum (*Sciaenops ocellatus*) [57], [58] and in body color regulation in medaka [59].

**Figure 1:**
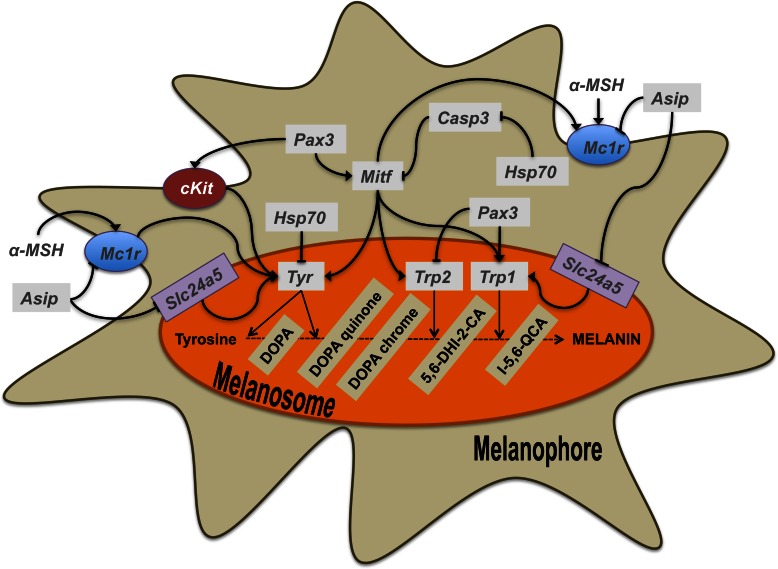
Model for the molecular action of the melanophore-differentiating and melanogenic genes within melanophores. Inside melanosomes, the master regulator of melanogenesis, *mitf*, can regulate the action of *tyr*, *trp2* and *trp1,* coding for the main enzymes responsible for the synthesis of melanin. Besides, the action of *Slc24a5,* which is the calcium melanosomal transporter, is crucial for proper melanin synthesis. *Pax3* is a key upstream transcription factor in the cascade that can promote or inhibit melanogenesis through transcriptional regulation *mitf* and *cKit*, the latter being necessary for melanophore differentiation and responsible for the activation of *tyr*. *Pax3* can also modulate the expression of the two other melanogenic enzymes *trp1* and *trp2*. *Mc1r*, located to the melanophore membrane, is activated by *α-MSH* and promotes the activation of *tyr*. *Asip* can inhibit the action of *mc1r* and *slc24a5*. *Hsp70* has been shown to be a negative regulator of *casp3* and the latter a negative regulator of *mitf*. *α-MSH, α*-melanocyte-stimulating hormone; *asip, agouti signaling protein*; *casp3, caspase 3*; *cKit, mast/stem cell growth factor receptor Kit; dtc/trp2*, *L-dopachrome tautomerase; mc1r, melanocyte-stimulating hormone 1 receptor*; *mitf*, *microphtalmia-associated transcription factor*; *pax3*, *paired box protein Pax-3; sl, somatolactin*; *slc24a5, sodium/potassium/calcium exchanger*; *tyr, tyrosinase*; *trp1, tyrosinase-related protein 1*.

The present study analyses, for the first time in fish, the expression profile of the above mentioned key genes involved in melanophore differentiation and melanogenesis during the larval development in an integrative physiology approach. Figure 1 shows a schematic design for a better visualization of the action of those genes within melanophores. Increasing the knowledge of the morphological and molecular ontogeny of skin pigmentation would help for a better understanding of this process in vertebrates and of the origin of pigmentation disorders. Indeed, fish have been used as models for melanoma research because it has been shown they share molecular signatures and histopathological features with human cancers [60].

## Results

### Growth, Survival, Pigmentation and Metamorphosis

Senegalese sole grew adequately throughout the larval stage, weighing 1.92±0.14 mg of dry weight and having a standard length (SL) of 8.75±0.13 mm at the end of the experimental period (47 dph). Survival rate and pigmentation success was 97.3±0.15 and 99.1±0.3%, respectively. The progress of metamorphosis based on eye migration [61] occurred within the following periods: pre-metamorphosis (until 11 dph), pro-metamorphosis (from 11 to 19 dph) and post-metamorphosis (from 19 to 47 dph).

### Morphological Ontogenesis of Skin Pigmentation

**Figure 2:**
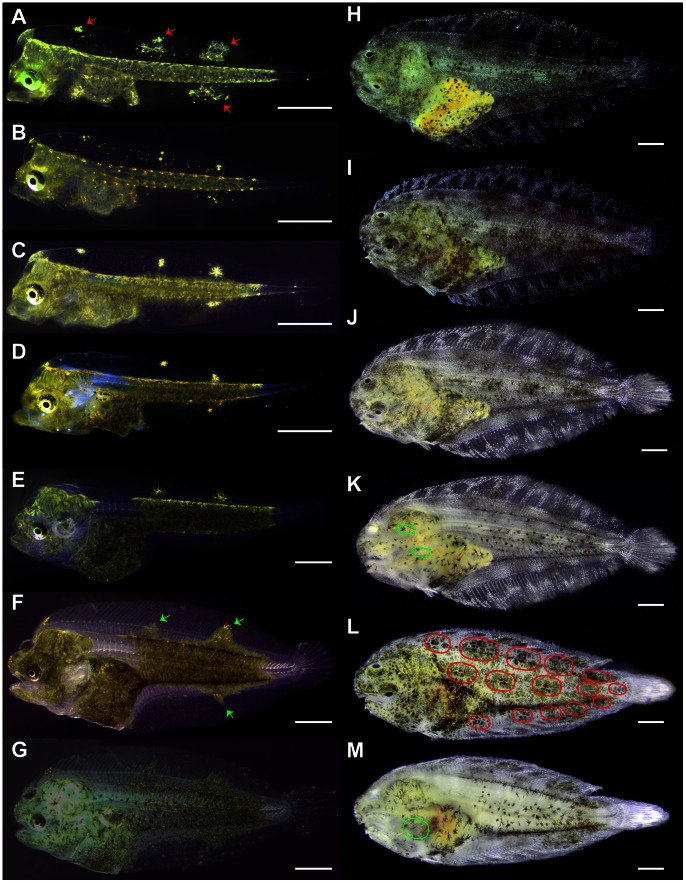
Morphological ontogeny of skin pigmentation in Senegalese sole larvae. A) 2 dph, B) 5 dph, C) 11 dph, D) 16 dph, E) 19 dph, F) 22 dph, G) 27 dph, H) 33 dph, I) 35 dph, J–K) 41 dph, L–M) 47 dph. Red arrows indicate patches of leucophores and xanthophores. Green arrows show patches of leucophores, xanthophores and melanophores. Note how the allocation of leucophores and xanthophores in dorsal and anal fins at very early stages of larval development serves as referring point for melanophore migration from the dorsal and ventral flanks of the fish to the fins. Red circles show 3 stripes of 5 patches of chromatophores conforming the juvenile pattern of skin color in the ocular side of the fish. Clusters of iridophores delimit these patches of melanophores, xanthophores and leucophores. Fins also follow the same distribution of chromatophores. Green circles delimit the area where iridophores were found in the skin of the blind side of the fish. Scale bar, 600 µm.

**Figure 3:**
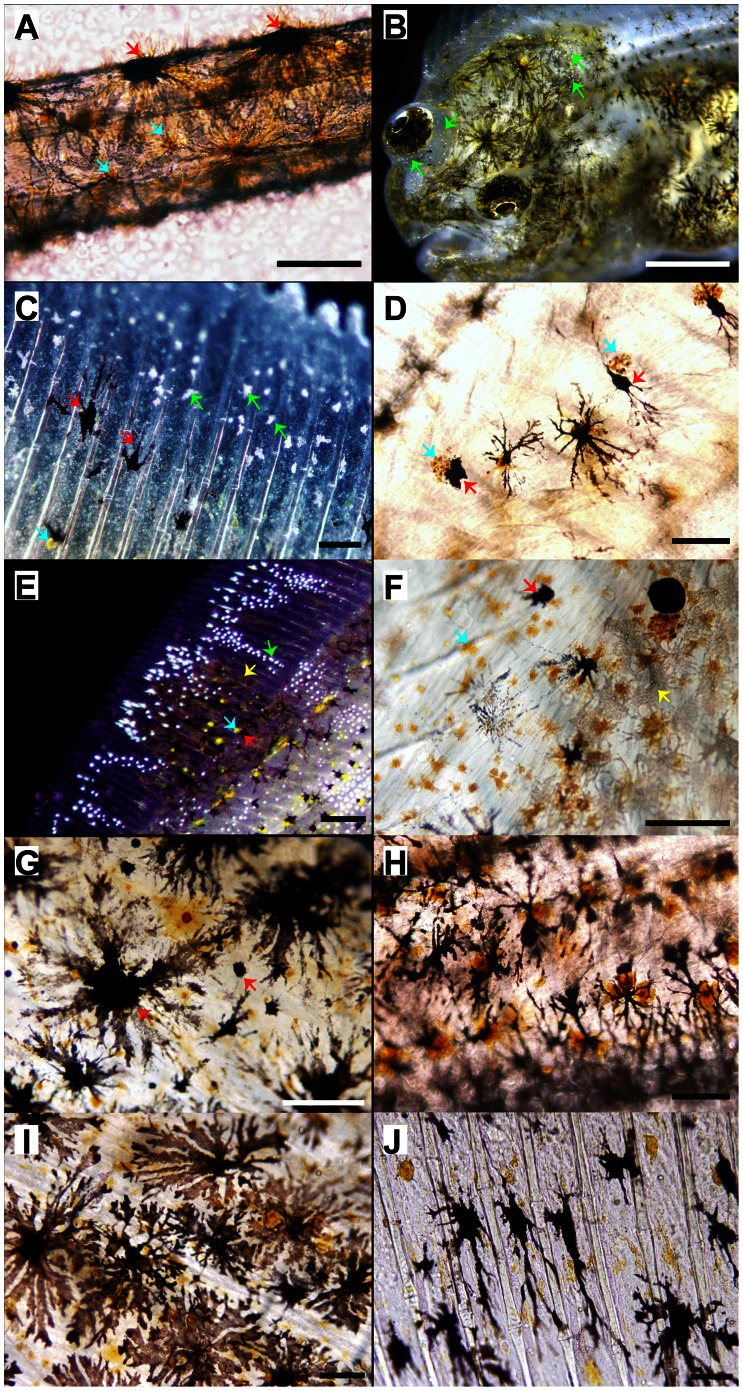
Images of the skin of Senegalese sole revealing the presence, shape, patterning and spatial relationships among melanophores, xanthophores, leucophores and iridophores. A) 5 dph, B) 22 dph, C–G) 33 dph, H) 35 dph, I-), 40 dph. A) Epidermal melanophores and xanthophores covered the dorsal and ventral flanks of the fish. B) Iridophores were already present in the skin of the ocular side of the fish at the level of the head. C) Detail of the dorsal fin showing melanophores, xanthophores and iridophores. D) Detail of the skin showing the interaction between xanthophores and melanophores. Note how communication between these cells leads to the disintegration of xanthophores. E) Detail of the distribution of chromatophores in the fins. A patch of melanophores, xanthophores and leucophores is surrounded by iridophores. F–J) Detail of the trunk skin of the ocular side showing the distribution pattern of xanthophores and melanophores. Note that the amount of xanthophores relative to melanophores decreased from 33 to 41 dph (F, J). Red arrows, melanophores; blue arrows, xanthophores; yellow arrows, leucophores; green arrows, iridophores; B, E, stereoscopic images; A, C–D, F–J, microscopic images. Scale bars: A, C, G, F, 200 µm; D, H, 250 µm; I, J, 100 µm; E, 500 µm; B, 1 mm.

**Figure 4:**
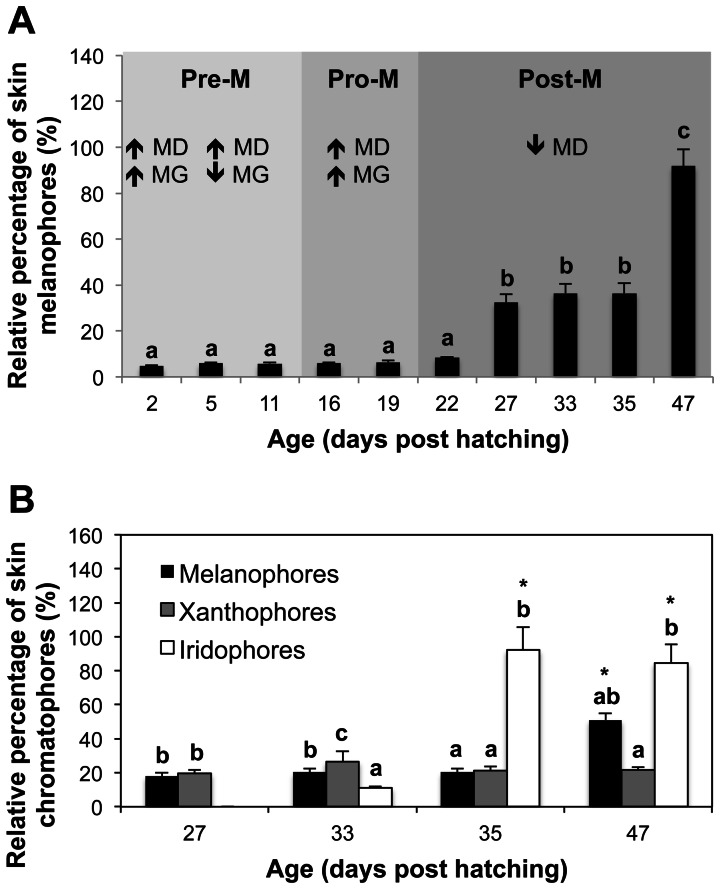
Relative amounts of chromatophores (in %) during the ontogeny of the ocular skin pigmentation in Senegalese sole. A) Relative amount of skin melanophores associated to the processes of melanophore differentiation and melanin synthesis governed by tightly controlled molecular signatures (see also Figs. 6 and 7). Values are expressed as mean ± SD (n = 4). Superscript letters denote significant differences among larvae of different age (One-way ANOVA, *P*<0.05). Pre-M, premetamorphosis; Pro-M, pro-metamorphosis; Post-M, post-metamorphosis; MD, melanophore differentiation [overall responses of *pax3*, *mitf*, *mc1r*, *cKit*], MG, melanogenesis [overall responses of *tyr*, *trp1*, *slc24a5*, *asip*]. B) Relative amount of skin melanophores, xanthophores and iridophores during post-metamorphosis. Values are expressed as mean ± SD (n = 4). Superscript letters denote significant differences between the relative amounts of chromatophores for a given larval age and asterisks indicate significant differences in the amount of a given chromatophore throughout the post-metamorphosis period (One-way ANOVA, *P*<0.05).

Morphological development of skin pigmentation in Senegalese sole is shown in Figs. 2 and 3. At 2 dph (3.07±0.02 mm), two lines of dendritic black melanophores (M), white leucophores and orange-yellowish xanthophores (X) overlaid the dorsal and ventral flanks of the body skin in the bilateral symmetric larva, with the exception of the future region of the caudal fin (Fig. 2A). These chromatophores also covered the skin of the head and abdominal area. Four patches of dendritic leucophores and xanthophores were located in the dorsal fin and one in the anal fin (Fig. 2A). The eyes were already pigmented at that time. One or several xanthophores associated to one melanophore could be seen three days later (Figs. 2B, 3A). Melanophores were located in the very surface of the skin and overlie the epidermis (Fig. 3A). This distribution of chromatophores remained similar until 11 dph (Fig. 2C); but round-shaped xanthophores were also observable and the patches of larval chromatophores located in the fins began to disappear at 16 dph (Fig. 2D). Metamorphosis was taking place and some larvae already showed a flattened body plan. At 19 dph, the linear pattern of allocation of the body skin chromatophores began to disorganize (Fig. 2E). At 22 dph, larvae were flat, although the eye from the blind side had not completely migrated to the ocular side (Fig. 2F). Some iridophores could already be seen in the head of some individuals (Fig. 3B). The relative amount of skin melanophores remained statistically invariable during pre- and pro-metamorphosis and represented an average of 6.20% (±1.23) of the maximum amount of melanophores quantified during the entire studied period (Fig. 4A).

Information about the developmental pattern of other chromatophores such as xanthophores and iridophores were only obtained during the post-metamorphosis period, since they were distributed in a dense and thick net that made difficult the proper recognition of single pigment cells at earlier stages of development. The same happened for leucophores and therefore they were not quantified. At 27 dph, the amount of iridophores increased and they began to organize to conform to the adult distribution pattern (Fig. 3C). In addition, the amount of melanophores significantly increased from 22 to 27 dph (Fig. 4A). The skin of the pro-metamorphic larvae contained similar amounts of xanthophores and melanophores, which represented, at this age, around 20% of the amount of pigment cells quantified during the studied period (Fig. 4B). Each melanophore was closely associated to one or two xanthophores (Table 1). Some xanthophores seemed to be disintegrating (Fig. 4D). The distribution of chromatophores was restricted to two bands on either side of the vertebral column and in the distal parts of the trunk, close to the beginning of the dorsal and anal fins. Some melanophores grouped to form a patch in the middle of the trunk. Two patches of chromatophores could be distinguished in the dorsal fin and another one in the ventral fin (Fig. 2G).

**Table 1:** Number of xanthophores (X) associated to one melanophore (M) during the post-metamorphosis period.

Age (dph)	X/M	N	SD
27	1.33^b^	4	0.65
33	4.75^ab^	4	2.43
35	1.81^b^	4	0.99
47	5.35^a^	4	2.20

Values are expressed in means ± SD. Superscript letters denote significant differences in the number of X related to one M between developmental ages (One-way ANOVA, *P*<0.05).

The migration of the left eye was completed in most larvae at 33 dph (Fig. 2H). On the ocular side, three lines of melanophores and xanthophores could be found in the dorsal and ventral trunk, from both sides of the vertebral column to the end of the trunk. These lines became discontinuous when a patch of iridophores was present. Then, patches of chromatophores, composed of a mixture of melanophores, xanthophores and leucophores, alternated with patches of iridophores, could be observed in the skin of the post-metamorphic larvae (Fig. 2H). There were five patches on the trunk, at the level of the vertebral column, five patches in the margin of the dorsal and ventral trunk, at the level of the proximal radials, and a higher number of patches, surrounded by iridophores, in the dorsal and ventral fins (Figs. 2H, 3E). This pattern of chromatophore distribution was preserved until the end of the studied developmental period (Fig 2L). Leucophores covered most of the trunk, where there were no iridophores present. In the dorsal and anal fins, leucophores were observed in the patches and also in the border of the dorsal fin (Figs 2H, 3E). At this time of development, two to seven xanthophores were associated with one melanophore (Fig. 3F–G, Table 1). Among the pigment cells quantified, xanthophores were the most abundant, followed by melanophores and finally by iridophores (Figs. 3F–G, 4B). From this time onwards, the shape of xanthophores was no longer dendritic, but round (Fig. 3F–H).

At 35 dph, the number of iridophores increased drastically, reaching the maximum relative percentage of ocular skin chromatophores quantified in this study (Figs. 2I, 4B), while the amount of melanophores and xanthophores became statistically equal again and still represented the same 20% of the total pigment cells counted at post-metamorphosis already observed 8 days earlier (Figs. 3H, 4B). The X/M ratio was around 2 (Table 1).

Between day 27 and 41 (Fig. 2J), the amount of melanophores in the skin of the ocular side remained invariable (Fig. 4A). However, the number of xanthophores decreased significantly at 41 dph with respect to that of melanophores and it was half the amount than at earlier stages (Fig. 3I–J, 4B). Between day 35 and the end of the study the amount of iridophores was higher than that of the other pigment cells (Fig. 2J, 4B). The blind side of the larvae was composed of melanophores and few xanthophores, and some iridophores could be observed at the level of the head (Fig. 2K).

At 47 dph, the pattern of skin pigmentation began to resemble that of adults (Fig. 2L). Chromatophores were organized in well-distinguished patches that covered the whole ocular side, including the head, trunk and fins. The amount of melanophores and xanthophores was higher than at 41 dph (Figs. 4A–B). There were no significant differences in the amount of melanophores and xanthophores, and the amount of iridophores was only higher than that of xanthophores (Fig. 4B). On the blind side, the skin was composed of a reduced number of melanophores, although there remained a few iridophores at the level of the head (Fig. 2M).

### Molecular Ontogenesis of Skin Pigmentation

**Figure 5:**
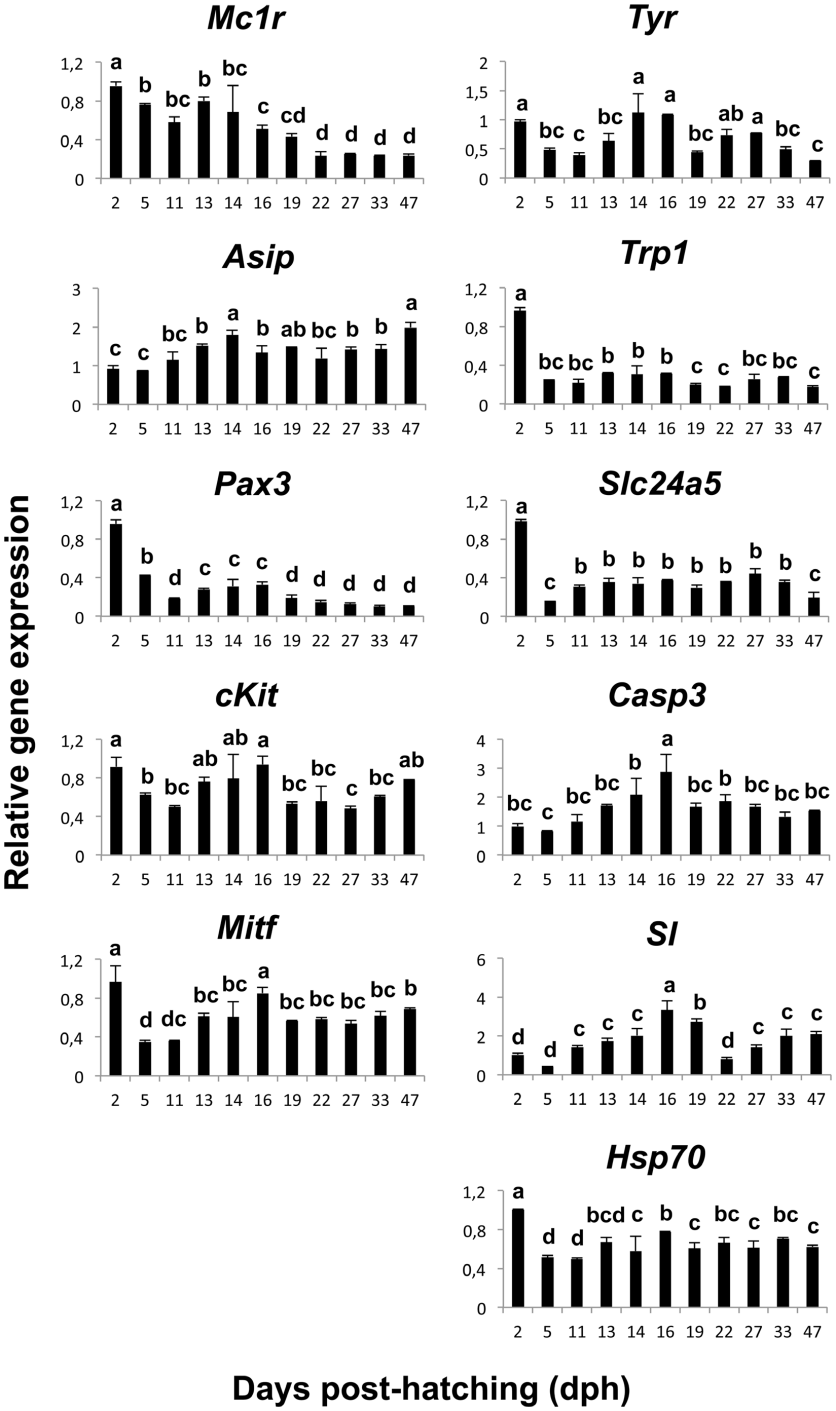
Gene expression patterns of pigmentation related genes during the larval development of Senegalese sole. *Melanocyte-stimulating hormone 1 receptor* (*mc1r*), *agouti signaling protein* (*asip*), *paired box protein Pax-3* (*pax3*), *mast/stem cell growth factor receptor Kit,* (*cKit*), *microphtalmia-associated transcription factor* (*mitf*), *tyrosinase* (*tyr*), *tyrosinase-related protein 1* (*trp1*), *sodium/potassium/calcium exchanger 5* (*slc24a5*), *caspase 3* (*casp3*), *somatolactin* (*sl*) and *heat shock 70 kDa protein* (*hsp70*) Data are represented as means of relative gene expression ± SD (N = 3). Values with a different superscript letter denote significant differences between sampling points (One-way ANOVA, *P*<0.05).

The expression patterns of the analyzed genes are represented in Fig. 5. The allocation of these genes to the melanogenesis pathway can be found in Fig. 1. *Mc1r* expression decreased significantly during development from 2 to 5 dph, from 5 to 16 dph and from 16 to 22 dph. The expression levels of *mc1r* remained constant from 22 dph onwards. *Asip* expression increased from 2 to 14 dph to slightly decrease at 16 dph and remained relatively constant until 33 dph. At 47 dph, the expression of *asip* increased reaching the levels observed at 14 dph. *Pax3* displayed the highest level of gene expression at 2 dph and then decreased until 11 dph (5 fold decrease). From 13 to 16 dph, *pax3* expression was 1.5 times higher than at 11 dph and subsequently decreased until 19 dph to remain stable onwards at the same levels of expression observed at 11 dph. *cKit* and *mitf* showed similar gene expression profiles during ontogeny. Again, the highest level of expression was detected at 2 dph followed by a decrease until 11 dph. Then, an increase in gene expression was observed between 11 and 16 dph. From 19 dph onwards, the expression level was constant and similar to that observed at 11–13 dph. *Tyr* displayed two peaks of expression, the first one between 14 and 16 dph and the second one between 22 and 27 dph, the level of expression being similar to that observed at 2 dph. *Trp1* displayed the highest level of gene expression at 2 dph. At 5 dph, *trp1* expression decreased 4 times and remained at the same level until 16 dph. Gene expression decreased again from 16 to 19 dph to remain invariable from that day until the end of the studied period. *Slc24a5* showed the highest level of expression at 2 dph. At 5 dph the amount of transcripts was 6 times lower than at 2 dph while at 11 dph it increased 2 fold with respect to 5 dph to remain at a constant level until 33 dph. At 47 dph, the expression level of *slc24a5* decreased to similar values observed at 5 dph. The expression level of *casp3* increased from 2 to 16 dph. The expression level decreased at 19 dph and remained invariable onwards. The amount of *sl* expression increased gradually from 2 dph reaching the highest level at 16 dph (8 fold increase). Subsequently, a 4-fold decrease of *sl* expression was observed from 16 dph to 22 dph. At 27 dph, *sl* increased 1.8 times with respect to day 22 and remain statistically constant afterwards. *Hsp70* showed the highest level of gene expression at 2 dph. From that time onwards, *hsp70* showed lower but fluctuating levels of expression.

**Figure 6:**
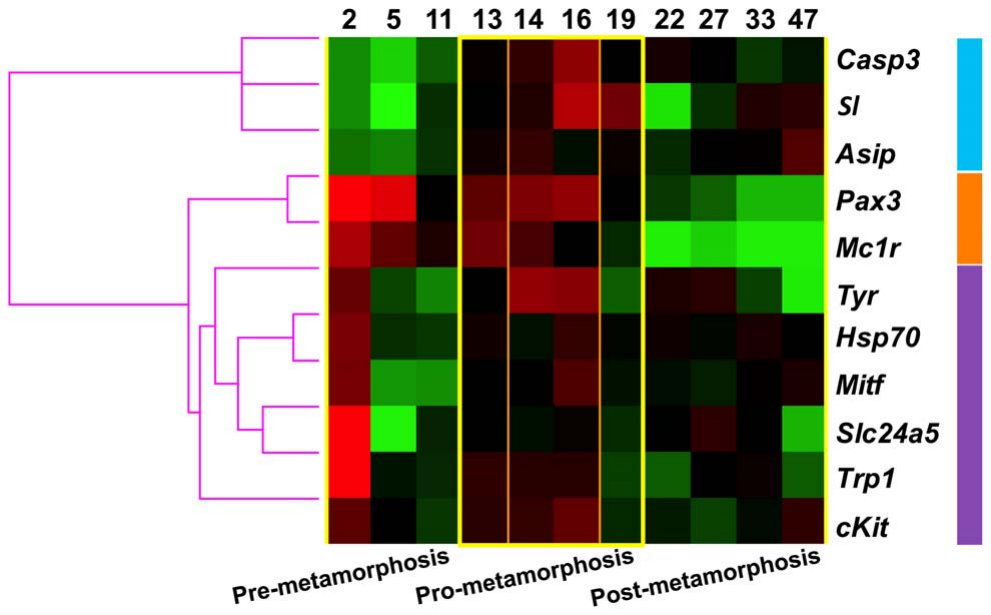
Global hierarchical clustering based on similarity of the expression profile for different pigmentation related genes during the larval development of Senegalese sole. Columns represent the mean data values for each sampling point (days post hatching) and rows represent single genes. Expression level of each gene is represented relative to its median abundance across the different stages and is depicted by a color scale: green, black, and red indicating low, medium, and high relative expression levels, respectively. Colored bars to the right margin indicate the three main gene clusters: blue shows genes highly expressed during pro-metamorphosis stage, orange corresponds to genes highly expressed during pre- and pro-metamorphosis and violet to genes highly expressed at 2 dph and at pro-metamorphosis. The three main stages of the larval development are indicated at the bottom of the figure. Note that the expression of all genes was high during the pro-metamorphosis phase (yellow square), most of genes displaying a shift in their level of expression before and/or after that period. The climax of pigmentation development at the molecular level was observed between 14 and 16 dph (orange square). Changes in gene expression profiles coincided with morphological changes in pigmentation, showing that the climax of metamorphosis was achieved at 16 dph and the end of pro-metamorphosis at 19 dph. The transition from the larval to the adult pattern of skin pigmentation could be clearly observed from 22 dph onwards.

Fig. 6 shows the global hierarchical clustering of genes based on their expression profile during the larval development. Gene clustering revealed two main groups. The first one included those genes displaying low levels of transcription at early stages of development (from 2 to 11 dph): *casp3*, *sl* and *asip*. The second cluster grouped the rest of the genes, which showed high levels of expression at 2dph. This cluster was divided into two main groups. The first one included *pax3* and *mc1r,* which were highly expressed during pre- and pro-metamorphosis (until 16–19 dph). The second group contained those genes displaying lower expression levels during pre-metamorphosis (5–11 dph). Within this group, *cKit* was placed alone whilst the other genes were grouped together. The latter grouping was divided in two clades. One composed of *tyr* and a second one that was divided again into two closely related sub-groups: *slc24a5* and *trp1,* and *mitf* and *hsp70*.

**Figure 7:**
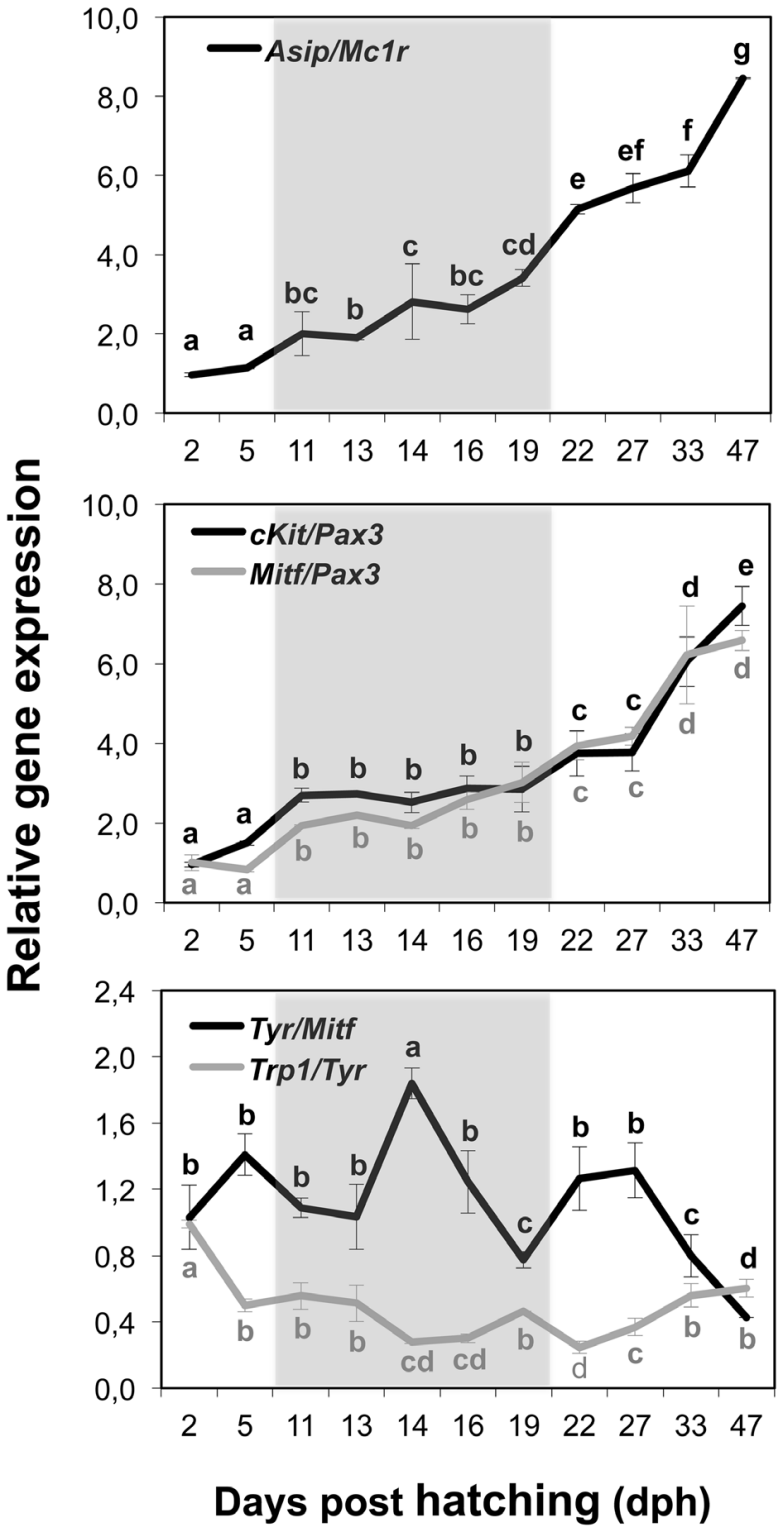
Gene expression ratios during the larval development of Senegalese sole illustrating the ontogeny of chromatophores. The *asip/mc1r* ratio shows the relationship between these genes and reflects the pigmentation patterning of Senegalese sole during development. Its increase during post-metamorphosis coincided with the switch from the bilateral to the flat symmetry and with the appearance of iridophores in the skin of the ocular side. The *cKit*/*pax3* and *mitf*/*pax3* ratios indicate the regulation of *cKit* and *mitf* transcription by *pax3.* Note the initial positive regulation of both genes by *pax3* and the subsequent stabilization during the pro-metamorphosis period. The increase of both ratios at post-metamorphosis shows the activation of melanophore migration and melanin synthesis processes during this period. The *tyr/mitf* and *trp1/tyr* ratios display opposite profiles during development and correspond well to the melanin production periods. The peaks of *tyr/mitf* ratio reveal the activation of the melanin synthesis pathway, whereas those of *trp1/tyr* ratio show the production of melanin. Grey regions indicate the pro-metamorphosis stage.

Fig. 7 shows *asip/mc1r, cKit/pax3, mitf/pax3, tyr/mitf* and *trp1/tyr* gene expression ratios, which allowed determining the different stages of metamorphosis in terms of pigmentation ontogeny. *Asip/mc1r* ratio increased slightly from 2 to 19 dph (y = 0.40x+0.54; r = 0.96) and then it sharply increased from that day onwards (y = 1.11x+2.45; r = 0.96) (ANCOVA, F = 11.64; P = 0.002). The *cKit/pax3* ratio increased from 2 to 11 dph, remained stable from 11 to 19 dph (pro-metamorphosis period), and increased again from 19 to 47 dph (One-way ANOVA, P<0.001). Similarly, the *mitf/pax3* ratio showed a staggered increase from 5 to 11 dph, from 19 to 22 dph and from 27 to 33 dph (One-way ANOVA, P<0.001). *Tyr/mitf* ratio presented two peaks at 14 and 22–27 dph (One-way ANOVA, P<0.001), whereas *trp1/tyr* decreased from 2 to 14 dph, then a peak was observed at 19 dph to subsequently decrease at 22 dph, and it increased gradually again from 22 to 33 dph (One-way ANOVA, P<0.001).

## Discussion

The regulation of pigmentation in vertebrates is a quite complex process that encompasses the migration of crest-derived stem cells during the embryonic development, their proliferation in target tissues (i.e., skin) and differentiation into mature chromatophores, and a tightly controlled regulation of melanogenesis. Pigmentation biology has been extensively studied and many aspects are today well known, especially in melanocytes [62], [63]. However, less is known about the molecular basis of melanogenesis due to the intricate network of pathways regulating this process that includes many multifunctional mechanisms of action [64]. Due to the similarities shared with humans, fish have become extremely valuable experimental animal models for vertebrate developmental studies, especially those with underlying genetic components [60]. The genetics of pigmentation have been explored in several model teleost fish including zebrafish [5], [65], medaka [66], fugu [67], goldfish [35], [68] and, recently, in flatfish [27], [69]. To our knowledge, this is the first study that combines the characterization of the morphological ontogeny of skin pigmentation with the analysis of the expression profile of a set of key pigmentation-related genes during the larval development of a fish.

It has been proposed that the various fish chromatophores differentiate from a common chromatoblast precursor [5]. This has been supported by recent research on the molecular mechanisms underlying cell fate decisions and differentiation in goldfish [35] and zebrafish [30], [39]. Distinct populations of melanophores and iridophores contribute to pigment patterns before and after metamorphosis [5], [70], [71], [72]. This issue has remained unresolved for xanthophores because of the lack of mutants able to ablate these cells [3], though the existence of two different populations, the “larval xanthophores” and the “adult xanthophores” has been recently suggested [27]. In the present study, changes in the expression of pigmentation related genes were found to be coincident with changes in pigment pattern of Senegalese sole. These changes occurred during pro-metamorphosis (Fig. 6) and morphological changes in the population of melanophores, xanthophores and iridophores were evidenced at post-metamorphosis leading to the adult pattern of pigmentation (Fig. 4B).

### Morphological Ontogeny of Skin Pigmentation

Pigment cells were already present in the skin of 2 day-old Senegalese sole larvae. Abundant larval-type xanthophores and melanophores (large dendritic cells) were distributed along the head, abdominal and trunk regions, with the exception of the caudal fin. Xanthophores were often associated to melanophores (Fig. 3A). The eye was also pigmented at that time (Fig. 2A). This developmental stage was in synchrony with the maturation of the main digestive organs and the mouth and anus opening, which took place at around 3 dph [73]. At this time, pigmented eyes are crucial for the larvae to be able to succeed in the transition from the endogenous feeding (yolk-sac reserves) to the exogenous feeding period, as prey search and capture mainly involves the vision during the larval stage [74].

The amount of melanophores and xanthophores remained invariable during pre- and post-metamorphosis in Sengalese sole larvae and were equally represented until 33 dph and, from that day to 41 dph, the amount of xanthophores decreased by a half. In contrast, the density of xanthophores decreased in Japanese flounder (*Paralichthys olivaceus*) before metamorphosis and increased rapidly after metamorphosis [9]. The population of round-shaped xanthophores became more abundant than the dendritic ones during post-metamorphosis and became the main cell type from 33 dph onward (Fig. 3F–H). An increase in the number of xanthophores, similar to that found in Japanese flounder [9], was detected at 47 dph in Senegalese sole. In Japanese flounder and stone flounder (*Kareius bicoloratus*) the same morphological type of xanthophore was observed before and after metamorphosis [24]. However, recent studies suggested also the existence of two different populations of xanthophores for Japanese flounder [27]. Similarly in this study, another population of melanophores appeared at post-metamorphosis (ca. 33 dph) (Fig. 4A) in Senegalese sole, coinciding with the increased amount of melanophores quantified in the ocular side during this period. An increase in the number of melanophores after metamorphosis was also observed in flounders [9], [24]. Previous studies on flounders have identified two distinct populations of melanophores that appeared sequentially during development [75] and differed in size [24]. In Senegalese sole larvae, two groups of melanophores were also observed according to their size (Fig. 3G). However, because those smaller melanophores were not found again until 47 dph (Figs. 2L, 4A), it more likely seems that they corresponded to the morphological stage of newly differentiated and melanized cells rather than the adult-type melanophore described for Japanese flounder. The newly formed small melanophores observed at 47 dph accounted for the increase in the amount of melanophores quantified at that date (Fig. 4A). Melanophores organized in patches in the ocular side of the fish at the end of pro-metamorphosis, whereas the blind side presented few melanophores mainly located in stripes along the margins of the dorsal and ventral flank and in the center of the trunk, and in the abdominal area. Iridophores on the ocular side of Senegalese sole first appeared at the end of pro-metamorphosis and increased in number and distributed in patches thereafter. In Pleuronectiformes (Flounders), iridophores also increased in number from metamorphosis and were restricted to patches in the ocular side while remaining distributed dispersedly in the blind side [9], [24]. The appearance of iridophores on the blind side was delayed to the late juvenile stage in Japanese flounder [9]. Unfortunately, the extension of the experimental period was not long enough to monitor the ontogeny of iridophores in the blind side of Senegalese sole, since only a few cells could be observed at the level of the head beginning at 41 dph. Another pattern of iridophore allocation was observed in plaice (*Pleuronectes platessa*) were these cells were the most prominent chromatophore on both sides of the body after metamorphosis [76]. Leucophores were present in the skin of Senegalese sole at early stages of development. During metamorphosis, leucophores contributed to the conformation of the adult pigmentation pattern by distributing in patches along the trunk and fins. Conversely to what occurred in Japanese flounder [27], leucophores did not disappear after metamorphosis in Senegalese sole. Morphologically, changes in pigment cells types and distribution occurred after metamorphosis in Senegalese sole.

These changes in number and distribution of chromatophores in the skin seem to not occur in an independent manner, but a kind of cellular communication exists between them enabling the pigmentation pattern to mature in a manner characteristic for each species. In this respect, it has been reported that xanthophores regulate melanophore pattern formation [6] and that melanophores are required for proper iridophore disposition in the skin of zebrafish [70]. These findings suggested the existence of a cascade of interactions among chromatophores in zebrafish: xanthophores −> melanophores −> iridophores [6]. A similar mechanism of pigment cell interaction seemed to occur in Senegalese sole larvae. After metamorphosis, xanthophores were more abundant than melanophores and closely associated to them, this being in line with the proposed role of the xanthophores in the guidance for melanophore patterning. Yamada et al. [6] demonstrated that pigment cell precursors migrate from the dorsal and ventral margins of the flank to the lateral sides of the body and to the dorsal and ventral fins in Japanese flounder to differentiate into adult-type pigment cells. Similarly, the patches of xanthophores and leucophores observed at 2 dph in Senegalese sole where located at specific points of the dorsal and anal fins and they seemed to function as reference points for the migration of melanophores to the fins at pro-metamorphosis (Fig. 2A–F) [77]. From 33 to 35 dph, a decrease in the proportion of xanthophores versus melanophores was found, with morphological evidences of disintegrating xanthophores (Fig. 2D). Then, from 35 dph, the number of xanthophores associated to one melanophore (X/M ratio) varied during post-metamorphosis, switching from 1 to 5 (Table 1). Concomitantly, the proportion of melanophores versus iridophores decreased and the distribution of iridophores was restricted to those areas free of melanophores (Figs. 2F–L, 3E, 4B).

Nevertheless, interaction between xanthophores and melanophores could be bidirectional. Xanthophores are able to eliminate the surrounding melanophores over a short-range in zebrafish [78]. Similarly, xanthophores in the skin of Senegalese sole pseudo-albinos seemed to be responsible for the degeneration of melanophores [Darias et al., unpublished data]. Considering this, analysis of the proportion of melanophores versus xanthophores during the development of Senegalese sole could be a suitable biomarker to evaluate the correct ontogeny of skin pigmentation in this species. Changes in the proportion of these pigment cells are undoubtedly preceded by changes in the molecular signaling. Identification of genes responsible for these specific types of patterning is necessary as a possible means for early detection of pigmentation disorders.

The differences found in the ontogeny of skin pigmentation between Senegalese sole and flounders reveal the complexity of the processes regulating pigmentation within the group of flatfish. Although metamorphosis of Senegalese sole was accomplished largely before the final sampling point, an extended experimental time would be necessary to completely describe the ontogeny of pigmentation in this species. This points to species-specific studies being necessary to find out the mechanisms underlying pigmentation that, in turn, will allow understanding the origin of pigmentation disorders in each species.

### Molecular Ontogeny of Skin Pigmentation

The morphological features of skin pigmentation ontogeny were mirrored in their molecular features (Figs. 2, 5, 6, & 7). As evidenced by the high level of expression of the pigmentation-related genes and the presence of abundant melanophores and xanthophores at 2 dph in Senegalese sole, molecular signaling toward differentiation of these cells occurred even earlier, including the process of melanin synthesis within the larval retina [79] (Figs. 5, 6). The expression patterns of *pax3*, *mitf* and *cKit* followed similar trends. This observation is in line with the known influence of *pax3* in the transcriptional modulation of the other two genes [38], [80]. *Pax3* and *mitf* play a key role in the differentiation of neural crest-derived melanocytes [36]. *Pax3* is also crucial for xanthophore differentiation in zebrafish. Knockdown of *pax3* resulted in a loss of xanthophores and an increase of melanophores, providing more evidence to the existence of a common chromatoblast precursor [39]. Indeed, *Pax3* is considered a xanthophore specification gene, whereas *mitf* was described earlier as a melanophore specification factor [39]. *cKIT* receptor influences melanogenesis, proliferation, migration, and survival of the pigment-producing cells [42] (Fig. 1). The expression of *mitf* and *cKit* increased progressively during pre- and pro-metamorphosis in Senegalese sole reflecting the differentiation and proliferation of melanophores (Figs. 1, 5, 6, & 7). Within the biosynthetic pathway of melanin, TYR catalyzes the rate-limiting conversions of tyrosine to DOPA, DOPA to DOPA-quinone and possibly 5,6-dihydroxyindole to indole-5,6 quinone. *TRP1* is involved in the oxidation of 5,6-dihydroxyindole-2-carboxylic acid (DHICA) into indole-5,6-quinone-2-carboxylic acid [80] (Fig. 1). The decrease in the amount of chromatophore differentiating *pax3*, *mitf* and *cKit*, and of melanogenic, *tyr* and *trp1,* transcripts between 16 and 19 dph indicated the climax of pigmentation development, and the beginning of the formation of the adult pattern of pigmentation (Figs. 2, 5, 6, & 7). These changes in gene expression coincided with the end of the pro-metamorphosis period. This is in agreement with the fact that, in mammals, *pax3* is expressed in early development, but inhibited in adult melanocytes [81], and *cKit* induces *tyr* expression during melanogenesis, but not in mature melanocytes [42]. Considering the gene expression pattern of *pax3* during ontogenesis and its role in the regulation of xanthophore and melanophore differentiation, this gene could be a potential candidate for monitoring the correct development of skin pigmentation in Senegalese sole. The *cKit/pax3* ratio in Senegalese sole reflected the ontogeny of melanophores as demonstrated by an increase in melanogenesis during pre-metamorphosis and a stabilization of melanophore differentiation and melanogenesis processes during pro-metamorphosis. Once metamorphosis was finished, the *cKit/pax3* ratio increased again until the end of the larval period indicating the prevalence of melanogenesis over melanophore differentiation. Similarly, the *mitf/pax3* ratio increased in a staggered way and could be indicating changes in the proportions of melanophores versus xanthophores.

In addition, the expression profile of *mc1r,* involved in the formation of pigmentation pattern during development, decreased during development until 19 dph to remain constant afterwards, indicating the end of melanophore ontogeny and pigment patterning at the transcriptional level. In fact, the stabilization of the gene expression levels of *pax3*, *mitf*, *cKit* and *mc1r* observed at post-metamorphosis coincided with the onset of the adult pigmentation phenotype (Figs. 2, 5, & 6).

The expression of the melanogenic genes *tyr* and *trp1* peaked asynchronously during the larval development, where peaks of *tyr* expression preceded peaks of *trp1* expression (Fig. 7). The changes in the *tyr/mitf* and *trp1/tyr* ratios clearly showed a cyclic production of melanin in this species during development. Moreover, the opposite profile of both ratios suggests the existence of a regulatory mechanism between *tyr* and *trp1* transcription in Senegalese sole (Fig. 7). Indeed, *TRP1* has been demonstrated to be a critical enzyme for the correct trafficking of *TYR* to melanosomes [82]. The first peak of *trp1* expression likely reflected the melanin production in larval melanophores, including the retina (2 dph) and the second one could be responsible for the melanin synthesis in the newly differentiated melanophores (19 dph). Therefore, the transition from pro- to post-metamorphosis was also evidenced by the expression profile of melanogenic genes. These results are in line with the thought that new melanophores differentiate from their precursors during metamorphosis [24]. A new peak of *tyr* expression was observed at 22 dph, followed by a peak of *trp1* expression 10 days later, suggesting a new population of melanophores was being stimulated into differentiation. This was corroborated by the increase in the amount of melanophores observed at 47 dph (Fig. 4A).

*Slc24a5* is a putative cation exchanger localized to intracellular membranes of melanosomes and their precursors and is believed to increase uptake and accumulation of calcium in melanocytes, necessary for melanogenesis [50], [51]. *Slc24a5* expression is necessary for melanin production in human epidermal melanoblasts stimulated to differentiate [83]. This explains the importance of a constant expression of this gene during the larval development of Senegalese sole. In particular, *Slc24a5* is required for *TRP1* protein expression in humans [83] and seems to be the same for Senegalese sole, as both genes were clustered together (Fig. 6).

In mammals, *ASIP* inhibits the *α-MSH/MC1R* signaling resulting in the production of phaeomelanin (yellow pigment) instead of eumelanin (brown/black pigment). Moreover, *ASIP* is able to inhibit the differentiation and proliferation of melanoblasts [84]. In rodents, *Asip* is expressed only in skin whereas in humans it has a wider pattern of expression, including adipose tissue, testis, ovary, and heart and lower levels of expression in foreskin, kidney, and liver (see review from [83]) and the physiological role, including hair and skin pigmentation, is not fully understood. In goldfish, *asip* is mainly expressed in the ventral skin and it is thought to be involved in the establishment of the dorsal-ventral pigment pattern by directing chromatophore differentiation, causing production of iridophores (structural pigment cells) and inhibiting production of melanophores [35]. *ASIP* has similar role in quails and chickens [85]. In Senegalese sole larvae, the *asip/mc1r* ratio remained constant during pro-metamorphosis and then increased considerably at post-metamorphosis, coinciding not only with the switch from the bilateral to the flat symmetry, but also with the increase in the amount of iridophores in skin of the ocular side of the fish (Figs. 2, 4, & 7). The results obtained in the present study show that the establishment of both the new dorsal-ventral body plan and skin pigment pattern is synchronized. Considering the roles of *mc1r* and *asip* in pigmentation, the *asip/mc1r* ratio could be considered as an indicator of the ontogenesis of iridophores and also as a marker for changes in the pigmentation pattern during development in Senegalese sole larvae (Figs. 2, 4, & 7). The use of whole larvae for gene expression analyses omitted the possibility to determine whether *asip* was expressed in the skin of the ocular and/or blind sides of Senegalese sole larvae. However, considering that very few iridophores were observed in the blind side during the analyzed period, it is tempting to speculate that *asip* could have different roles at the same time on both sides of the fish. For instance, to first promote iridophore differentiation only on the ocular side while blocking melanin synthesis within melanophores of the blind side, as a step preceding development of iridophores in the blind side (Fig. 1). In fact, *asip* expression increased again at 47 dph to the levels observed at pro-metamorphosis, possibly indicating the beginning of iridophore development on the blind side (Fig. 5). Matsumoto and Seikai [24] already suggested that differentiation of adult melanophores at metamorphosis is blocked in the skin of the blind side of Japanese flounder. Moreover, it has been shown that an excess of *asip* gene expression could be responsible, at least in part, for the pseudo-albinism in Senegalese sole juveniles [Darias et al. unpublished data] by causing the down-regulation of *slc24a5* expression and, consequently, of *trp1* expression, thus preventing melanogenesis. This “altered” molecular mechanism occurring in the ocular side of pseudo-albinos could be “normally” happening in the blind side of the well pigmented larvae. Gene expression analyses using isolated skin samples from the ocular and blind sides could be used to test such a hypothesis.

It has been recently demonstrated that melanophores and iridophores are derived from a common precursor in zebrafish and the differentiation is driven by a *foxd3*/*mitfa* transcriptional switch; the role of *foxd3* being both to promote iridophore development and block melanophore differentiation by repressing *mitfa* [30]. Results of these authors suggested that cell precursors expressing *mitf* are bi-potent, therefore plastic, so those continuing to express this gene become melanophores, while others will repress *mitf* to form iridophores. Matsumoto and Seikai [24] observed that the most common pigment cells derived from cultured chromatoblasts *in vitro* are iridophores and that the precise moment for differentiation of melanophores responsible for the adult coloration is genetically programmed. Whether these cells have a common precursor in Senegalese sole is unknown, but the molecular (*asip*/*mc1r* ratio) and morphological data indicates that chromatoblasts are first differentiated into melanophores. According to [30], the decrease of *mitf* gene expression from 16 to 19 dph could indicate a higher number of bi-potent cells differentiate into iridophores on the ocular side and there is a blockage in melanophore development on the blind side (Figs. 2M, K, 5).

During metamorphosis of Senegalese sole, many physiological functions, especially growth [86], [87], [88], are slowed down and stored energy reserves are consumed in switching from bilateral symmetry to the typical body asymmetry of flatfish, including the eye migration and skull remodeling. This study revealed a transcriptional up-regulation of all genes analyzed during the phase of pro-metamorphosis showing that the transition from larval to juvenile pigmentation is also in synchrony with that process (Fig. 6). The expression profile of *casp3* indicated that cell apoptosis during larval development of Senegalese sole was especially active during the pro-metamorphosis period. Similar profiles were observed for *asip* and *sl.* The gene expression profile of *asip* is in line with its recently described involvement in the up-regulation of genes that are normally expressed during morphogenesis [89]. *Sl* is a member of the growth hormone/prolactin family, and has been shown to enhance differentiation of light-absorbing pigment cells (melanophores and xanthophores) and suppress differentiation of light-reflecting cells (leucophores and iridophores) in medaka [59]. In Senegalese sole larvae, the expression profile of *sl* was in line with the sequential development of melanophores and iridohores. Gene expression of *pax3* and *mc1r* was also high during pre-metamorphosis, indicating their early implication in chromatophore differentiation, particularly melanophores and xanthophores, and patterning. A third gene cluster observed included those highly expressed at 2 dph and which later slowed down during pre-metamorphosis. These genes were involved in chromatophore differentiation and melanogenesis. The high level of *hsp70* expression observed at 2 dph seems to be coherent with the expression profile of *casp3* since it has been shown that HSP70 protects WEHI-S cells from CASPASE-3-induced cell death [90]. However, most of the stem cells on the blind side of Japanese flounder appeared to undergo cytolysis without evidences for apoptosis [75], [91].

Altogether these observations revealed different stages of skin pigmentation and development in Senegalese sole that coincided with the progress of metamorphosis. These stages could be summarized as follows: 1) pre-metamorphosis period (2–11 dph): low expression of apoptotic factor and genes related to melanogenesis (with the exception of day 2), and high expression of melanophore differentiating genes; 2) pro-metamorphosis period (11–19 dph): high expression of apoptotic factors (tissue remodeling) and melanophore differentiating and melanogenic genes; 3) post-metamorphosis (19–47 dph): low expression of all analyzed genes, especially those associated to melanophore differentiation (Figs. 4A, 6).

Senegalese sole larvae already presented at 2 dph around 6% of the maximum amount of melanophores counted within the studied period, which remained invariable until 22 dph (Fig. 4A). This result illustrates the precocious action of the molecular mechanisms governing pigmentation and explains the high levels of expression of melanophore differentiation- and melanogenic-related genes observed at 2 dph. Interestingly, the expression of melanogenic genes was lower during the rest of the pre-metamorphosis period. Considering that the amount of melanophores remained invariable during pre- and pro-metamorphosis and that larvae already possessed pigmented melanophores (Fig. 2), melanogenesis begins quite early. Because melanophore differentiation occurred during both pre- and pro-metamorphosis periods, the increase in the expression of melanogenic-related genes observed at pro-metamorphosis was likely an indicator of melanin synthesis within the new developed melanophores. Likewise, the increased expression of melanophore differentiation-related genes during pro-metamorphosis resulted in an increase in the amount of melanophores during post-metamorphosis. The increased population of melanophores could induce the down-regulation of melanophore differentiating-related genes. Indeed, the relative amount of melanophores significantly increased by 23% at 27 dph and remained constant at post-metamorphosis, which was supported by the low expression of melanophore differentiation-related genes observed after day 27 (Fig. 4A). Altogether these results have shown that alternating actions of both melanophore differentiation- and melanogenesis-related genes coordinates melanophore ontogenesis in Senegalese sole. The *cKit/pax3*, *tyr/mitf*, *asip/mc1r* and *trp1/tyr* ratios reflected well these molecular events (Fig. 7), suggesting that quantification of the expression of these genes could be a useful tool to evaluate the state of the pigmentation process during early development of Senegalese sole.

While ontogenesis of skin pigmentation in Senegalese sole is genetically programmed, environmental factors can modulate the normal changes in the molecular processes occurring during metamorphosis. However, these alterations are morphologically detectable only after metamorphosis [Darias et al., unpublished data]. Knowledge of the molecular mechanisms underlying flatfish pigmentation may help to explain the synergy between genetic, behavioral and environmental influences on this process, aiding in understanding the appearance of pigmentation problems in other vertebrates, including humans. Nutrition is one of the factors affecting pigmentation in flatfish (reviewed by [92]). Previous studies have demonstrated that high levels of dietary arachidonic acid induced pseudo-albinism in developing Senegalese sole [25]. Therefore, nutritional approaches can be suitable for analyzing the origin and mechanisms of some types of pigmentation disorders during the ontogeny of fish. Flatfish are particularly well-suited to this since, as we have seen in this study, morphologic markers have been identified which correspond to underlying molecular mechanisms that are at work during the ontogeny of pigmentation. Future efforts need to focus on contrasting the differences in gene expression that occur in the ocular and blind sides of developing larvae.

## Methods

### Ethics Statement

This study was carried out in accordance with the recommendations in [94]. Animal experimental procedures were conducted in compliance with the experimental research protocol (reference number 4978-T9900002) approved by the Committee of Ethic and Animal Experimentation of the IRTA and the Departament de Medi Ambient i Habitatge (DMAH, Generalitat de Catalunya, Spain) in accordance with EU regulation (EC Directive 86/609/EEC).

### Animal Rearing and Sampling Procedures

Senegalese sole larvae were obtained from Stolt Sea Farm SA (Carnota, La Coruña, Spain), acclimated at IRTA-SCR facilities and reared at 17.0±2.8**°**C and 35 of salinity in four 500 l cylindrical tanks (initial density: 50 larvae l^−1^) connected to an IRTAmar™ recirculation unit. Water was renewed daily (20%) with gentle aeration in each tank, pH and dissolved oxygen being 8.0±0.2 and 7.5±1.3 ppm, respectively. Photoperiod was 16L: 8D, and light intensity was 500 lx at the water surface. Larvae were fed twice a day, from 3 to 10 dph, with rotifers (*Brachionus plicatilis*) enriched with Easy Selco™ (ES, INVE, Belgium) following manufacture’s recommendations, at a density of 10 rotifers ml^−1^ from 3 to 6 dph and of 5 rotifers ml^−1^ from 7 to 10 dph. *Artemia* metanauplii enriched with ES were supplied to larvae from 6 to 37 dph at increasing density from 0.5 to 12 metanauplii ml^−1^. *Artemia* metanauplii density was adjusted four times per day (at 9, 12, 15 and 18 h) according [93] to assure the optimal prey density. From 33 dph to the end of the experiment (47 dph), larvae were progressively weaned onto dry feed (Gemma Micro 150–300^©^ Skretting, Spain). Eight larvae were sampled from each tank at 2, 5, 11, 16, 19, 22, 27, 33, 35, 41 and 47 dph to study the morphological development of skin pigmentation. For gene expression analyses, 200 mg wet weight larvae (80 to 3 individuals per sample time depending on fish size) were sampled at 2, 5, 11, 13, 14, 16, 19, 22, 27, 33, and 47 dph, sacrificed with an overdose of anesthetic (Tricaine methanesulfonate, MS-222, Sigma), rinsed in distilled water and preserved in RNAlater© (Ambion) at −80**°**C for further analyses. Fish were sacrificed following the protocols of the veterinary committee of IRTA in accordance with EU regulations (EC Directive 86/609/EEC).

### Photography and Image Analysis

Alive not anesthetized larvae were examined under stereomicroscope (Nikon SMZ800, Soft Imaging Systems, GmbH) and photographed using a Color View-XS camera at 300 dpi. Skins of larvae were photographed using a DP70 (Olympus) camera attached to DMLB (Leica) microscope. Images were taken under transmitted or incident light and compiled and processed using Analysis®3.1 (Soft Imaging Systems, GmbH). Types of pigment cells were classified by coloration and shape. ImageJ64 software was used to quantify the number of melanophores, xanthophores and iridophores. During pre- and pro-metamorphosis periods, the number of melanophores was quantified in the skin of the left side body trunk of the larvae, excluding the abdominal region. The relative amounts of xanthophores and iridophores were only quantified during the post-metamorphosis period because at earlier stages they were distributed in a dense and thick net that made difficult the proper recognition of single pigment cells. The same happened for leucophores and therefore they were not quantified. During the post-metamorphosis period, when individuals showed flat symmetry, the amount of melanophores, xanthophores and iridophores was quantified in the ocular side of the trunk skin, excluding the abdominal area. Results were represented as the relative proportion, expressed in percentage, of each chromatophore in the analyzed skin area.

### Real Time PCR Assays

Eleven genes involved in the process of pigmentation were selected as markers for melanophore differentiation and melanin synthesis in Senegalese sole larvae (Table 2). The ontogeny of gene expression was analyzed at 2, 5, 11, 13, 14, 16, 19, 22, 27, 33 and 47 dph. Total RNA of a pool of larvae was extracted using TRIzol™ (Invitrogen) following the manufacturers recommended protocol. The quantity of isolated RNA was determined by measuring optical density at 260 nm, using a Gene-Quant spectrophotometer (Amersham Biosciences), and its purity was evaluated by the absorbance ratio 260/280 nm. RNA samples with a 1.9–2.0 ratio were used for reverse transcription. The quality of the RNA extraction was further examined visually using sample aliquots separated electrophoretically in 1.2% agarose gels. Total RNA (1 µg) from each sample was reverse-transcribed using QuantiTect Reverse Transcription Kit (Qiagen®, GmbH, Germany). Real-time PCR analysis was performed using an ABI PRISM 7300 (Applied Biosystems). Amplification reactions were performed in triplicate in a total volume of 20 µl containing 1 µl of cDNA, 1 µl of Taqman probe, 10 µl of Taqman mix and 8 µl of RNAse and DNAse free water. The gene ubiquitin (*ubq*) was chosen as reference gene since it did not exhibit any significant variation in expression levels among the samples. The amplification conditions were 10 min at 95°C and 40 cycles of 15 s at 95°C and 1 min at 60°C.

**Table 2:** Target genes used in this study with their accession numbers, main biological processes and amplicon size, primers and hydrolysis probes used in qPCR analyses.

Gene name	GenBank accession no.	Biological process/Activity	Amplicon size	Hydrolysis probes	5′ to 3′ sequence
*Ubq*	**AB291588**	Ubl conjugation pathway	86	Forward	GCCCAGAAATATAACTGCGACAAG
				Reverse	TGACAGCACGTGGATGCA
				FAM probe	ACTTGCGGCATATCAT
*Tyr*	**JF693907**	Melanin biosynthesis	73	Forward	CGTACGCACAGATGGAAAACG
				Reverse	CACGTAGTAATGCATCCACACAAAA
				FAM probe	ACATCGGCGAATATC
*Trp1*	**GU329041**	Melanin biosynthesis; Melanocyte differentiation	63	Forward	CGTGTGCAACAATACAGAAACAAGT
				Reverse	ATGGGTCGTGCCACGTT
				FAM probe	CCTGCCGGGTTCCTT
*Mitf*	**GU329042**	Transcription factor for tyr, trp2 and trp1	75	Forward	CGATGACATCATAAGTCTTGAATCCAGTTT
				Reverse	CGTGCTGGGCAACTGAAGA
				FAM probe	CCGGAGTCAATCAACG
*cKit*	**HM100237**	Tyrosine-protein kinase signaling pathway	69	Forward	GTGAAGAGAGTGAGATGTTTGACGA
				Reverse	CACTTTGGTAGGAGAAGCTCAGAA
				FAM probe	CTCGTCACCGAAGATC
*Mc1r*	**GU329043**	Melanocyte-stimulating hormone receptor activity	76	Forward	CGCCGTCGCCATCATC
				Reverse	GCGTTGTCCGTGTGGTAGA
				FAM probe	ACCTCCAGCATCCTCT
*Scl24a5*	**GU329046**	Ion transport in melanosomes	66	Forward	GACGCAGCCTCTGATCGA
				Reverse	CCGTCCTGGAGCGAACC
				FAM probe	CCAGTCTGCGAAACAT
*Casp3*	**GU329040**	Apoptosis	77	Forward	CGACAGTGTAGATGACCAAACGT
				Reverse	GGAGCAGTGGAATAAGCATAAAGGA
				FAM probe	CCTCCACAGGAATCC
*Pax3*	**HM100238**	Transcriptional regulation of pigmentation	68	Forward	GCATCATGCGCTCCAAGTTC
				Reverse	CCCTCTTCACCATTTCATCATCCT
				FAM probe	CATCGTCACCAACTCC
*Sl*	**U06753**	Hormone activity	75	Forward	TTCCCACTGCGGCTTCA
				Reverse	GGTAAGGCCTTGGTGATGCA
				FAM probe	CCGACCGTGTTTCTC
*Asip*	**HE598753**	Regulation of melanogenesis	81	Forward	GCTGTGACATCTGTGCCTTCT
				Reverse	CCATTCGACAGAAACACACAGTTC
				FAM probe	CCAGTGTCGCCTCTTC
*Hsp70*	**GU329044**	DNA repair	73	Forward	TGGAGTCGTATGCTTTCAACATGA
				Reverse	TGCTTGTCGTCGTCACTGAT
				FAM probe	CTTGCCAGCCAGTTTC

Custom Taqman® assays were designed and provided by Applied Biosystems, Life Technologies (Table 2). The average efficiency of amplification for all assays was 99%. Real-time PCR efficiencies were determined for each gene from the slopes obtained with Applied Biosystems software, applying the equation E = 10[−1/slope], where E is PCR efficiency.

To determine the relative quantity of target gene-specific transcripts present in the different samples, expression ratios were calculated according to Pfaffl’s mathematical model [95]. Individuals of 2 dph were chosen as reference sample. Expression patterns of genes were visualized using supervised hierarchical clustering [96] applied on samples classified according to developmental stage.

### Calculations and Statistics

All changes to chromatophore percentages were normalized by comparison to a baseline value for iridophores, which were chosen as they were the most abundant. Changes in the percent of chromatophores and melanophores (Fig. 4) were calculated using the quantity of iridophores counted on day 35 as a baseline, giving consideration to this date of development as the time when adult pigment patterning is normally developed. Results were expressed as mean ± SD (n = 4). All data were checked for normality (Kolmogorov-Smirnov test) and homogeneity of variance (Bartlett’s test) and transformed when necessary. One-Way ANOVA was performed to analyze differences in the relative number of chromatophores and gene expression. When significant differences were found (*P*<0.05), the post-hoc Holm-Sidak method was used to perform all pairwise multiple comparisons. Statistical analyses were conducted using SigmaStat 3.0 (Systat Software Inc., Richmond, USA). ANCOVA was used to compare the slope of *asip*/*mc1r* ratio before and after metamorphosis of Senegalese sole (*P*<0.05).
